# Pharmacological combination of simvastatin and doxorubicin reduces tumor growth and lung metastasis in triple-negative breast cancer

**DOI:** 10.3389/fcell.2026.1807269

**Published:** 2026-07-28

**Authors:** Monserrat Llaguno-Munive, Wendy Hernández-Hernández, Erika Solano-Ibañez, María del Pilar Ramos-Godínez, Adriana Méndez-Bernal, Rafael Jurado, J. Omar Muñoz-Bello, Patricia Garcia-Lopez

**Affiliations:** 1 Laboratory of Pharmaco-Oncology, Basic Research Division, National Cancer Institute, Mexico City, Mexico; 2 Laboratory of Medical Physics, Basic Research Division, National Cancer Institute, Mexico City, Mexico; 3 Department of Surgical Pathology, National Cancer Institute, Mexico City, Mexico; 4 Department of Pathology, Faculty of Veterinary Medicine and Animal Husbandry, National Autonomous University of Mexico, Mexico City, Mexico; 5 Laboratory of Epidemiology and Molecular Biology of Oncogenic Viruses, Basic Research Division, National Cancer Institute, Mexico City, Mexico

**Keywords:** doxorubicin, drug repurposing, histopathology, lung metastasis, simvastatin, triple negative breast cancer

## Abstract

**Introduction:**

Triple-negative breast cancer (TNBC) is the most aggressive subtype of breast cancer, lacking expression of estrogen, progesterone, and HER2 receptors, limiting the efficacy of targeted therapies. Patients with TNBC face a poor prognosis, with a median survival of less than 5 years. Drug repurposing has emerged as a promising strategy to improve therapeutic options and overcome resistance in TNBC.

**Methods:**

We evaluated the antitumor efficacy of simvastatin, a cholesterol-lowering drug, in combination with doxorubicin using MDA-MB-231 TNBC cells and spheroids, and an orthotopic mouse TNBC model. The cells and spheroids were treated with doxorubicin either individually or in combination with simvastatin to assess the effects on viability, migration, and invasion. *In vivo*, tumor-bearing mice were administered doxorubicin (1 mg/kg, i.v.) and/or simvastatin (20 mg/kg, oral) twice weekly for 3 weeks. Primary tumor growth was monitored using a caliper and near-infrared fluorescence imaging (NIRF), while their metabolic activity was assessed by micro-PET/CT with 18F-fluorodeoxyglucose (18F-FDG). The pulmonary metastasis was assessed via NIRF imaging and histological analysis.

**Results:**

*In vitro*, the combination of simvastatin and doxorubicin significantly inhibited MDA-MB-231 cell proliferation, migration, and invasion, compared to either treatment alone; and in spheroids, the cytotoxicity was significantly decreased. *In vivo*, the doxorubicin/simvastatin combination significantly decreased the tumor growth rate and the pulmonary metastasis. Histopathological analysis of lung sections showed reduced neoplastic infiltration, and Ki-67 immunohistochemistry showed decreased cellular proliferation in the combination group.

**Conclusion:**

Our findings suggest that simvastatin enhances the therapeutic response of doxorubicin and may represent a valuable adjuvant strategy to control metastatic progression in TNBC.

## 1 Introduction

Triple-negative breast cancer (TNBC) is the most aggressive subtype of breast cancer, characterized by its high invasiveness and strong propensity to metastasize to critical organs, including the brain, lungs, liver, and bones. This subtype predominantly affects women under the age of 40 years and is associated with a poor prognosis, with an average survival of only 18 months after diagnosis, and represents 10%–15% of all breast cancer cases (Dent et al., 2007; Schmid et al., 2018; O'Reilly et al., 2021).

The lack of hormone receptors in TNBC makes antihormonal therapy and HER2-targeted drugs ineffective, which significantly limits therapeutic options. Current treatment mainly includes anthracyclines (such as doxorubicin and daunorubicin), taxanes (paclitaxel and docetaxel), capecitabine, and gemcitabine, among others (Fisusi and Akala, 2019). Doxorubicin is the most used drug for the treatment of TNBC; however, its use is limited by cardiotoxicity, as many women develop chronic cardiomyopathy. Treatment response rates to doxorubicin are around 40% when used as a single agent in metastatic breast cancer, due to these adverse effects, as well as drug resistance that can develop during treatment (Paridaens et al., 2000; Sledge et al., 2003). Therefore, there is an urgent need to develop new treatment strategies that enhance therapeutic efficacy while minimizing adverse effects.

Drug repurposing is a widely studied strategy for cancer treatment, which identifies new therapeutic applications for existing drugs that have already been approved for other therapeutic indications. This option has been widely utilized in recent years as an alternative to developing new drugs. Recently, drug repurposing supported by *in silico* studies has been implemented, and over 200 drugs with antineoplastic activity have been identified to date (Hua et al., 2022). Among the repurposed drugs for cancer, statins have recently shown promise in reducing cancer recurrence and affecting the proliferation and survival of cancer cells (Singhal et al., 2022).

Simvastatin has been widely used to reduce cardiovascular disease risk and manage high lipid levels. Its primary mechanism of action involves inhibiting endogenous cholesterol production by targeting the enzyme 3-hidroxi-3-metilglutaril-CoA reductase (HMG-CoA reductase). However, it has been reported that simvastatin could play a significant role in modulating pathways involved in cancer progression (Jiang et al., 2021; Nowakowska et al., 2021). Recent studies suggest that simvastatin significantly affects cancer cell migration by inducing cell cycle arrest at the G1 phase. Additionally, it reduces cellular invasion by downregulating matrix metalloproteinases (MMPs), which play a critical role in tumor progression and metastasis (Shen et al., 2015).

Therefore, combining therapeutic agents with repurposed drugs is a promising strategy for cancer treatment. In this study, we aimed to investigate the pharmacological effect of the combination of doxorubicin with simvastatin to evaluate its impact on lung metastasis and improve the response of the current treatment in triple-negative breast cancer. This study included preclinical assessments both *in vitro* (including cells and spheroids) and *in vivo* (using an orthotopic model of experimental TNBC) to evaluate whether the incorporation of simvastatin into the doxorubicin treatment regimen could improve therapeutic efficacy by inhibiting tumor growth and preventing metastasis, thereby improving the survival rates and quality of life for TNBC patients.

## 2 Materials and methods

### 2.1 Drugs and reagents

Doxorubicin was obtained from Sigma Chemical (Lot. 038k1349) (St. Louis, MO, United States), and a stock solution was prepared at a concentration of 2 mg/mL in sterile water. Simvastatin sodium salt was obtained from Enzo Life Sciences (Lot. M42LZ01) (Farmingdale, NY, United States); a stock solution was prepared at a concentration of 4 mg/mL in sterile water. Modified Eagle’s medium (DMEM), RPMI-1640 medium, FCS (fetal calf serum), EDTA (Ethylenediaminetetracetic acid), Tris, and SDS were obtained from Gibco, BRL (Grand Island, NY, United States). XTT assay was obtained from Roche Molecular Biochemicals. High-quality water employed to prepare solutions was obtained through a Milli-Q Reagent.

### 2.2 Cell culture

The triple-negative breast cancer cell line MDA-MB-231 was obtained from the American Type Culture Collection (ATCC, Rockville, United States) and cultured under sterile conditions in Roswell Park Memorial Institute 1640 (RPMI) medium supplemented with 10% fetal bovine serum (FBS) at 37 °C, with 5% CO2.

### 2.3 Cytotoxicity of simvastatin, doxorubicin, and their combination in the MDA-MB-231 cells

To evaluate the effect of Simvastatin on cell viability, MDA-MB-231 cells were seeded at a density of 9,000 cells per well in 96-well plates. After 24 h, the cells were exposed to various concentrations of doxorubicin alone (0–5 μM) or doxorubicin in combination with simvastatin (1, 2.5, and 5 μM) for 72 h. Control cells were exposed only to vehicles. After treatment, an XTT assay was performed. The experiments were conducted in three independent assays in duplicate.

### 2.4 Cell viability by XTT assay

Cell viability was assessed in the cultures using the Cell Proliferation Kit II (Cat. No. 11465015001): Sodium 3’-[1-(phenylaminocarbonyl)-3,4-tetrazolium]-bis(4-methoxy-6-nitro)benzene sulfonic acid hydrate) (XTT). Metabolically active cells convert XTT to the orange dye formazan. At the end of the treatment, the cells were incubated for 30 min with XTT at a final concentration of 0.3 mg/mL. Formazan produced by the metabolic activity of the cells was quantified with a spectrophotometer at 492 nm and 690 nm with a plate reader (Varioskan LUX, Thermo Scientific, Waltham, MA, United States). The percentage of viability was calculated, and the inhibitory concentration 50 (IC50) of doxorubicin alone or doxorubicin combined with simvastatin was calculated using GraphPad Prism 8.0.1 software (San Diego, CA, United States).

### 2.5 Wound healing scratch assay

To evaluate the effect of the drugs on cell migration, MDA-MB-231 cells were seeded in silicone culture-inserts 2 well (Ibidi No. cat 81176) in RPMI medium supplemented with 2% FBS. Cells were pre-treated with mitomycin C (20 μg/mL) for 30 min to inhibit cell proliferation. After, cells were exposed to doxorubicin alone (0.05 µM), simvastatin alone (1 µM), and doxorubicin and simvastatin in combination. The rate of wound closure was calculated at 0 and 24 h. ImageJ software (version 1.8.0) was used to calculate the wound closure rate.

### 2.6 Cell invasion assay

Invasion assay was assessed using Transwell chambers (Corning Inc., Corning, NY, United States) with an 8-μm pore size. The upper chambers were coated with matrigel (10 μg/cm^2^) and dried for 1 h at ambient temperature. MDA-MB-231 cells were exposed to doxorubicin alone (0.05 µM), simvastatin alone (0.1 µM), and doxorubicin and simvastatin in combination for 48 h. Following incubation, cells were trypsinized, and 2 × 10^5^ MDA-MB-231 cells were seeded in 100 μL of serum-free medium into the upper chamber; 600 µL of RPMI medium supplemented with 10% SFB was added to the lower chambers. Cells were incubated at 37 °C in 5% CO_2_ for 24 h. After incubation, non-invading cells were removed from the membrane surface using a cotton swab. The membranes were fixed with 4% paraformaldehyde for 15 min and stained with 0.1% crystal violet for 30 min. The stained cells were observed under a microscope at ×10 magnification.

### 2.7 Cytotoxicity of simvastatin and doxorubicin in a 3D culture (spheroids)

For spheroid formation, 4,000 MDA-MB-231 cells/well were seeded into a 96-well ultra-low attachment plate (Nunclon Sphera, Thermo Scientific, Denmark) in Essential 8 Basal Medium (E8) (Gibco by Thermo Fisher Scientific). The plate was centrifuged at 600 *g* for 3 min, then incubated for 24 h at 37 °C in a 5% CO2 atmosphere to form spheroids. After 3 days of cell seeding, the spheroids with an average size of 350 µm were exposed to different treatments for 5 days: doxorubicin alone (0.25 μM), simvastatin alone (2 μM), or a combination of both drugs. Each condition was evaluated in triplicate in three independent experiments (9 spheroids per condition).

### 2.8 LIVE/DEAD staining in spheroids

At the end of the treatment, we evaluated the live and dead cells within the spheroids using the LIVE/DEAD Viability/Cytotoxicity Kit for Mammalian Cells (Thermo Fisher Scientific). The process followed the manufacturer’s recommended protocol. Briefly, the spheroid samples were incubated with the staining reagent (calcein AM 2 μM and ethidium homodimer-1 (EthD-1) 4 μM) for 20 min at 37 °C and 5% CO2. After incubation, the spheroids were washed with PBS, placed on slides, and observed using a fluorescence microscope (OLYMPUS IX51). Two filters were used for imaging: 520 nm for calcein (live cells) and 590 nm for ethidium homodimer (dead cells). The images were subsequently processed and merged using ImageJ software to quantify the live and dead cells.

### 2.9 Viability assay in spheroids

Following the 5-day treatment, spheroids were transferred to a luminescence plate (Corning Incorporated, NY, United States). Cell viability was assessed using the CellTiter-Glo® 3D Cell Viability Assay (Promega Corporation, Madison, WI, United States), according to the manufacturer’s protocol. This assay is a method for determining the number of viable cells in 3D cell culture by quantifying ATP, a marker of metabolic activity. Addition of the CellTiter-Glo® 3D Reagent directly to cells results in cell lysis and generation of a luminescent signal proportional to the amount of ATP present. Briefly, 50 µL of reagent was added to each well, the plate was shaken at 400 rpm for 10 min, and incubated at room temperature for 20 min. Luminescence was measured using a microplate reader (VARIOSKAN LUX, Thermo Fisher Scientific Inc.), and viability graphs were generated based on data from three independent experiments in triplicate (9 spheroids per condition).

### 2.10 Transfection of MDA-MB-231 cells with the piRFP plasmid

To enable the monitoring of tumor growth using near-infrared fluorescence (NIRF) imaging, stable transfections were performed in MDA-MB-231 cells with the piRFP (phytochrome-based near-infrared fluorescent protein) plasmid (Addgene 31,857) using Lipofectamine 2000 reagent (Invitrogen, Carlsbad, CA) following the manufacturer’s instructions. Forty-eight hours post-transfection, cells were selected with geneticin G418 (2 g/L) (G418, Santa Cruz Biotechnology, Heidelberg, Germany). After 30 days of selection with geneticin, clonal selection was performed to ensure the isolation of a single cell and generate a stable clone with identical genetic characteristics. Stable expression was achieved through antibiotic selection with geneticin (G418) sulfate (Santa Cruz Biotechnology, Inc., Dallas, TX), and stability of piRFP expression was routinely verified before *in vivo* experiments by assessing fluorescence signal and confirming expression stability.

The clones were characterized and isolated by flow cytometry using FACS (Fluorescence Activated Cell Sorting) (FACS ARIA II (PO241), Cytometry Unit of the National Institute of Respiratory Diseases, Mexico). The cells were maintained in RPMI medium supplemented with 10% fetal bovine serum (FBS) and geneticin (2 g/L) at 37 °C with 5% CO_2_.

### 2.11 *In vivo* assays

#### 2.11.1 Preclinical breast cancer model with lung metastasis

Female NOD/SCID mice, age 8 weeks old, were provided by the Institute of Biomedical Research of UNAM, Mexico City, Mexico, and maintained under pathogen-free conditions, with *ad libitum* access to food and water, a 12-h light/dark cycle, a temperature of 27 °C, and appropriate humidity levels. The handling and sacrifice of the mice were conducted in accordance with the official Mexican Federal Regulations for Animal Experimentation and Care (NOM-062-ZOO-1999, Ministry of Agriculture, Mexico). The ethics, research, and animal care and use committees approved the protocol. This study adheres to internationally accepted standards for animal research, following the 3Rs principle. The ARRIVE guidelines were employed for reporting experiments involving live animals, promoting ethical research practices.

For the preclinical breast cancer model with lung metastasis, an orthotopic surgery was performed on mice, implanting MDA-MB-231 cells (4 × 10^6^ cells/50 µL) transfected with piRFP plasmid, into the mammary fat pad. Before surgery, the mice were anesthetized in a 2.5% isoflurane atmosphere. The second mammary area was disinfected with an aseptic solution, and an incision was made to expose the mammary tissue. Once identified, the mammary fat pad was inoculated with tumor cells, and the incision was closed with 3M Vetbond™ tissue adhesive. Tumors were periodically monitored after the inoculation. Two perpendicular diameters were measured using a caliper, and tumor volume was determined using the following equation: V = (π/6) × D × d^2 (D = large diameter; d = small diameter).

When the tumors reached a volume between 80–100 mm^3^, the mice were randomized into four groups (n = 5 per group): doxorubicin alone (1 mg/kg, administered intravenously, twice weekly for 3 weeks); simvastatin alone (20 mg/kg, administered orally, twice weekly for 3 weeks); or the combination of both drugs. The control group received only the vehicle (injectable water). Doxorubicin (Lot. 038k1349) was prepared at a concentration of 2 mg/mL in sterile water, and simvastatin sodium salt (Lot. M42LZ01) was prepared at a concentration of 4 mg/mL in sterile water.

The doses of simvastatin used (20 mg/kg) and doxorubicin (1 mg/kg) were in accordance with previous preclinical studies reported in the literature, which report efficacy and acceptable tolerability in murine breast cancer models (Watson et al., 2022; Mabrouk et al., 2023).

Tumor growth was monitored with a caliper and by near-infrared fluorescence (NIRF) imaging at 700 nm with the Odyssey® DLx Imaging System (LI-COR). NIRF imaging was performed at baseline (at the start of treatment), at the end of the treatment (day 21), and 1 week after the end of the treatment (day 28), and pulmonary metastasis was evaluated by NIRF imaging at a wavelength of 700 at the end of the experiment. Tumor and non-tumoral tissue Regions of Interest (ROIs) were manually segmented using the 700 nm channel. To estimate background fluorescence, ROIs of the same size were placed in adjacent non-tumoral tissue. The fluorescence signal was calculated by subtracting the background intensity from the fluorescence intensity of the tumor ROI from the same animal.

### 2.12 Tumor growth by molecular imaging using microPET-CT

The microPET-CT images were acquired at the end of the treatment (day 21), and 1 week after the end of the treatment (day 28), mice were administered 6 MBq of ^18^F-fluorodeoxyglucose (^18^F-FDG) via intravenous injection. One hour post-injection, animals were anesthetized with 2% isoflurane and subjected to molecular imaging using a microPET/CT scanner (Albira ARS, Oncovision, Spain). For PET/CT images, ROIs were drawn on fused PET/CT images in PMOD (software version 4.2, PMOD Technologies Ltd., Switzerland), using the CT anatomical frame to ensure reproducible tumor segmentation. The resulting images were reconstructed and analyzed using PMOD 4.2.

NIRF imaging and PET/CT images were exported and renamed using a randomized coding scheme before analysis. Tumor ROIs were manually delineated by one trained investigator who was blinded to treatment assignment.

### 2.13 Evaluation of pulmonary metastasis

Two weeks after the end of treatment, the animals were euthanized by exposure to carbon dioxide (CO_2_) following the AVMA Guidelines for the Euthanasia of Animals. Cervical dislocation was performed immediately after respiratory arrest to confirm euthanasia. Their lungs were extracted for NIRF imaging analysis using the Odyssey® DLx Imaging System at 700 nm (LI-COR). For lung metastasis analysis, a lesion was considered positive when the fluorescence signal was approximately 60 fluorescence units above the background signal and was clearly distinguishable from the surrounding tissue (n = 4-5 animals per group).

### 2.14 Histology and immunohistochemistry

The animals were euthanized, and the lungs were perfused with saline solution followed by 4% paraformaldehyde. The lung tissue was embedded in paraffin and sliced into sections (2 mm thick) on the coronal plane for the subsequent analysis with Eosin and Hematoxylin (H&E), and cell proliferation was examined according to the level of the Ki-67 protein. Histological and immunohistochemical analyses were performed on lung tissue from three animals per group.

Quantification of the Ki-67 index was performed using ImageJ software. For each sample, ten fields were analyzed. The Ki-67 proliferation index was calculated as the percentage of positive nuclei (brown staining) to the total number of nuclei. All analyses were performed in a blinded manner using coded samples.

### 2.15 Statistical analysis

The statistical analysis of the results was performed using GraphPad Prism software version 8.0.1. A repeated-measures ANOVA test followed by a Tukey *post hoc* test was applied. Data were reported as mean ± SD or SEM, and a p-value < 0.05 was considered statistically significant. *In vivo* assay, animals were randomly assigned to different groups once tumors reached the predefined volume (∼80–100 mm^3^) (n = 4-5 animals per group). Investigators were blinded to treatment allocation during data acquisition and analysis. Investigators were also blinded during imaging acquisition, data analysis, and histopathological evaluation. Groups were blinded by assigning codes, and the analyst remained blinded until the statistical analyses were completed.

## 3 Results

### 3.1 Cytotoxicity of the combination of doxorubicin and simvastatin in MDA-MB-231 cells

**FIGURE 1:**
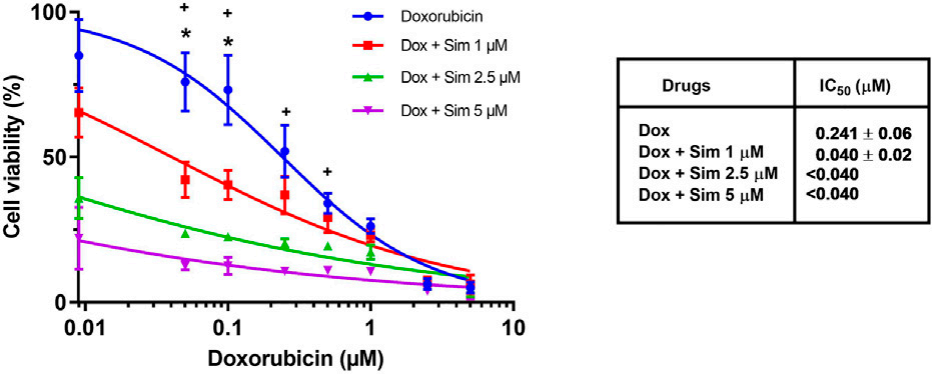
Dose-response curves for the combination of simvastatin and doxorubicin. Each data point represents the mean of three independent experiments in duplicate; results are expressed as mean ± SD (*) Statistically significant difference (p ≤ 0.05) between doxorubicin vs. the combinations (Dox + Sim 1 μM, Dox + Sim 2.5 μM, Dox + Sim 5 μM) (+) statistically significant difference (p ≤ 0.05) between Dox + Sim 1 μM vs. Dox + Sim 2.5 μM, Dox + Sim 5 μM.

The effects of simvastatin and doxorubicin on MDA-MB-231 cells exposed to different concentrations of the drugs alone or in combination for 72 h, are shown in Figure 1. A dose-dependent response was observed in the cell viability of the drugs. Furthermore, the effect of doxorubicin was significantly enhanced when combined with simvastatin. It was observed that increasing the concentration of simvastatin reduced the IC50 values of doxorubicin, demonstrating a more significant effect with 5 µM simvastatin, resulting in a greater decrease in doxorubicin IC50 from 0.24 to < 0.040 µM.

### 3.2 Wound healing migration and invasion assays

**FIGURE 2:**
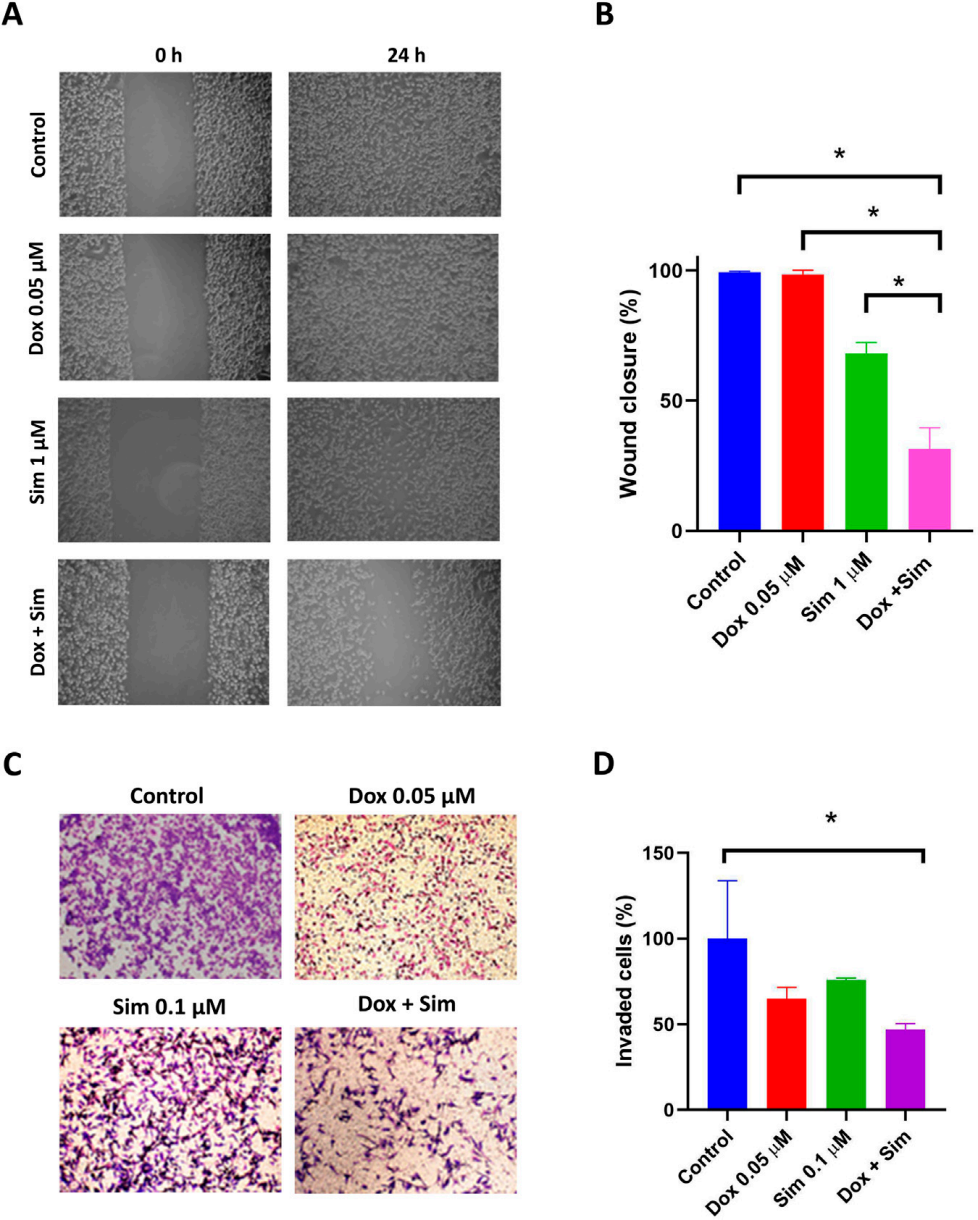
Effect of simvastatin and doxorubicin on migration and invasion in MDA‐MB‐231 cells. **(A)** Representative images of cell migration assays in MDA‐MB‐231 cells exposed to Dox 0.05 µM, Sim 1 µM, and the combination Dox+Sim after 24 hours. **(B)** Percentage of wound closure after 24 hours of exposure. Data represent the mean of three independent assays ± SD. (*) Statistically significant difference (p ≤ 0.05) between Dox+Sim vs control, Dox alone, and Sim alone. **(C)** Representative images of Invasion assay (n=3) after 48 hours of exposure to Dox 0.05 µM, Sim 0.1 µM, and the combination Dox+Sim. Cells were fixed and stained with crystal violet. **(D)** Quantification of invaded cells expressed as percentage relative to control. Data are presented as mean ± SD of three independent experiments (*p < 0.05).

A wound-healing scratch assay was performed to evaluate the effect of simvastatin and doxorubicin on cell migration. Figure 2A shows the images at 0 and 24 h; it was observed that the wound closed entirely in the control and doxorubicin groups. On the other hand, we observed that simvastatin alone inhibited cell migration; however, this inhibition was more pronounced in the combination group. Figure 2B presents the quantification of wound closure, showing a significant difference in the group with combined drugs compared to individual treatments. For the invasion assay (Figure 2C), MDA-MB-231 cells treated with doxorubicin exhibited a greater inhibition of invasion compared to the control group and cells treated with simvastatin alone. However, the combined treatment of simvastatin and doxorubicin enhanced the inhibition of cell invasion compared to either treatment alone. Quantification of invaded cells (Figure 2D) confirmed a significant reduction in invasion in the combination group.

### 3.3 Cytotoxicity of the combination of doxorubicin and simvastatin in 3D culture

**FIGURE 3:**
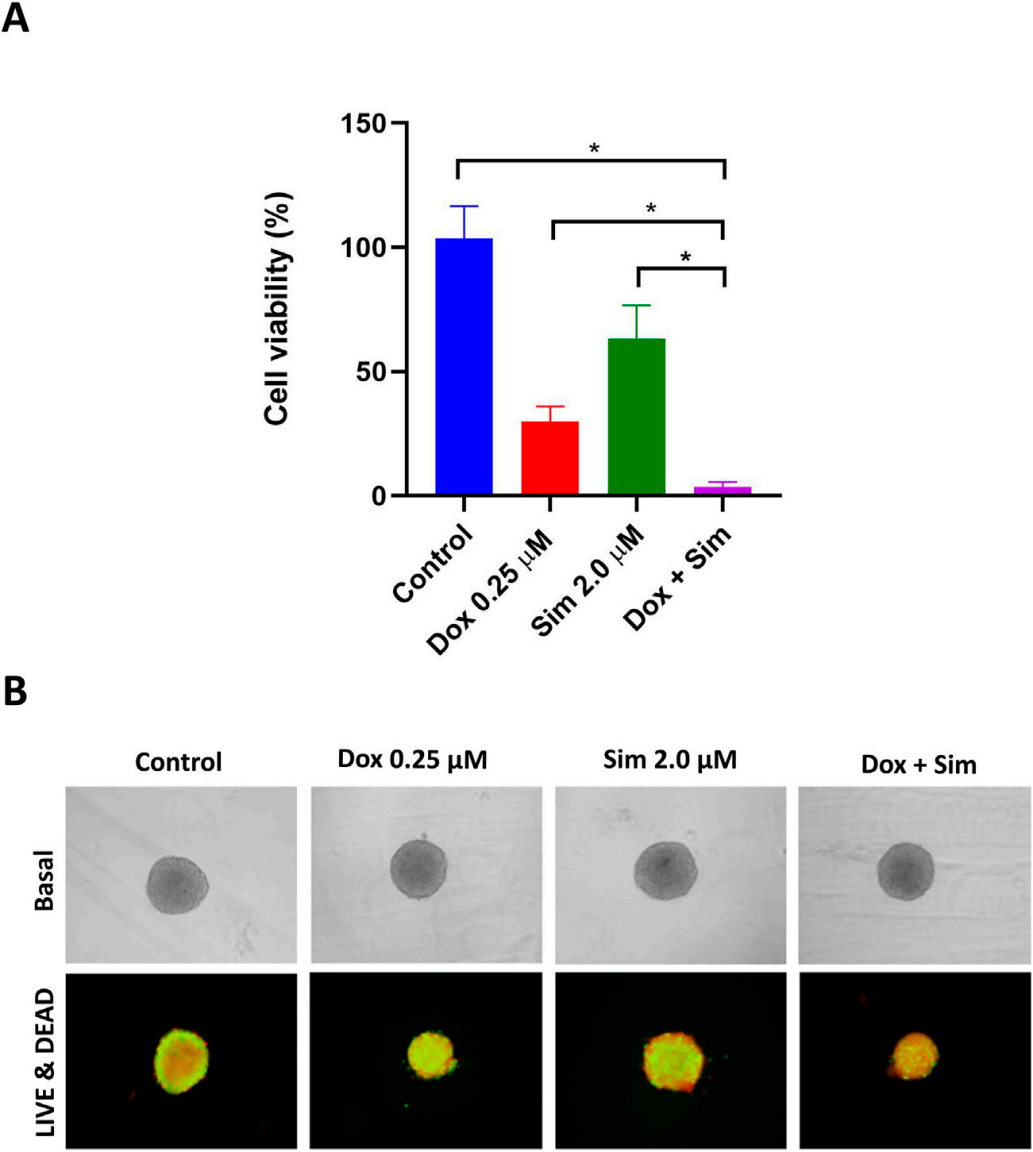
Evaluation of cell viability in spheroids treated with doxorubicin and simvastatin. **(A)** Quantification of cell viability after the 5-day treatment period. **(B)** Images of spheroids stained with calcein (live cells) and ethidium homodimer (dead cells). Data are presented as mean ± SD from three independent experiments in triplicate (9 spheroids per condition) (*p < 0.05).

The results of the cell viability with the CellTiter-Glo assay showed a significant decrease in cell viability in the spheroids treated with the combination of doxorubicin and simvastatin compared to the drugs alone (Figure 3A). These findings are corroborated by LIVE and DEAD assay, in which the control group and the groups treated with simvastatin and doxorubicin alone exhibited the highest calcein staining (green fluorescence), indicating a predominance of live cells (green fluorescence) compared to the combination of doxorubicin alone and simvastatin alone. In contrast, spheroids treated with the combination showed predominant staining with ethidium homodimer (red fluorescence) (Figure 3B).

### 3.4 Effect of doxorubicin and simvastatin on tumor growth in an orthotopic TNBC model

**FIGURE 4:**
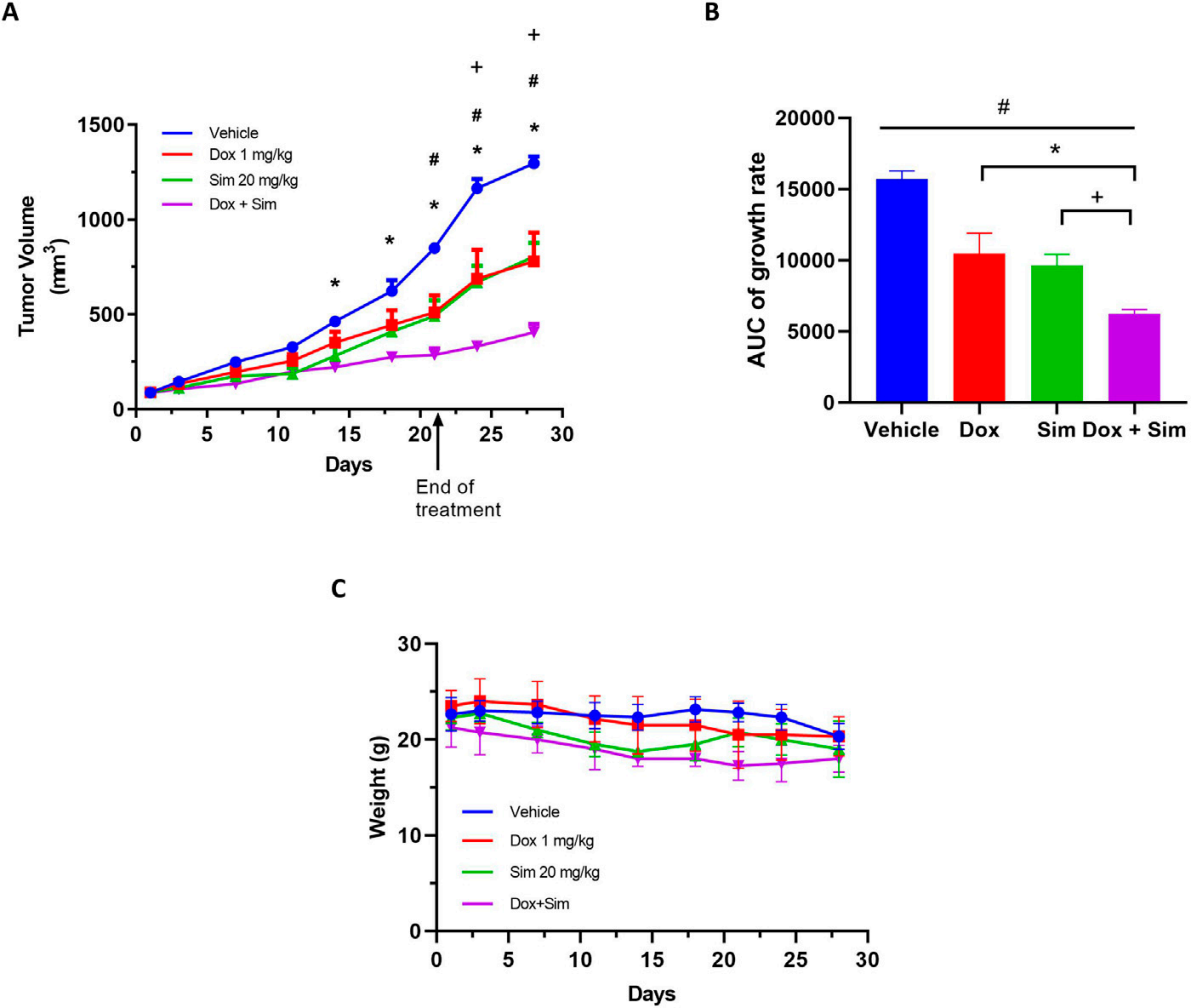
Antitumor activity on the orthotopic breast cancer model. **(A)** Tumor growth rate of different treatments: Vehicle, Doxorubicin (1 mg/kg), simvastatin (20 mg/kg), and the combination of doxorubicin + simvastatin. Each point represents the mean ± SEM of five animals (*) indicates a significant difference (p < 0.05) between the vehicle vs. the combination group (Dox + Sim) (#) indicates a significant difference (p < 0.05) between the vehicle and the groups treated with simvastatin or doxorubicin alone (+) represents a significant difference (p < 0.05) between the groups treated with simvastatin alone or doxorubicin alone vs. the combination group (Dox + Sim). **(B)** Area under the curve (AUC) for the tumor growth time course was calculated from the tumor volume vs. time plots. Each point represents the mean ± SD of five animals (#) indicates a significant difference (p < 0.05) between the vehicle and all treatment groups (*) indicates a significant difference (p < 0.05) between doxorubicin and the combination group (Dox + Sim), and (+) indicates a significant difference (p < 0.05) between simvastatin and the combination group (Dox + Sim). **(C)** Body weight monitoring throughout the experimental period to assess systemic toxicity. Data are expressed as mean ± SD (n = 5 animals per group).

Figure 4 shows the effects of simvastatin and doxorubicin alone and combined with simvastatin in an orthotopic breast cancer model. The animals treated with simvastatin and doxorubicin alone showed a decrease in tumor growth rate compared to the vehicle group. However, the group receiving the combination of doxorubicin and simvastatin demonstrated significantly greater control of primary tumor growth (Figure 4A). The area under the curve (AUC) of the tumor growth time course was calculated to obtain an integrated measure of cumulative tumor burden over the duration of the study (Figure 4B). By capturing global differences in growth dynamics rather than relying on isolated time-point comparisons, the AUC provides a more robust assessment of treatment efficacy. AUC values were computed from individual tumor growth curves using the trapezoidal rule. The results showed that the vehicle group differed significantly from all treated groups, while both doxorubicin and simvastatin alone showed significant differences with the combination treatment. These results support an interaction between doxorubicin and simvastatin in suppressing tumor growth. Body weight evolution was monitored as an indicator of systemic tolerance (Figure 4C). All treatments were well tolerated; no animals exhibited a reduction greater than 20% of their initial body weight.

### 3.5 *In vivo* molecular imaging of tumor response to treatments

**FIGURE 5:**
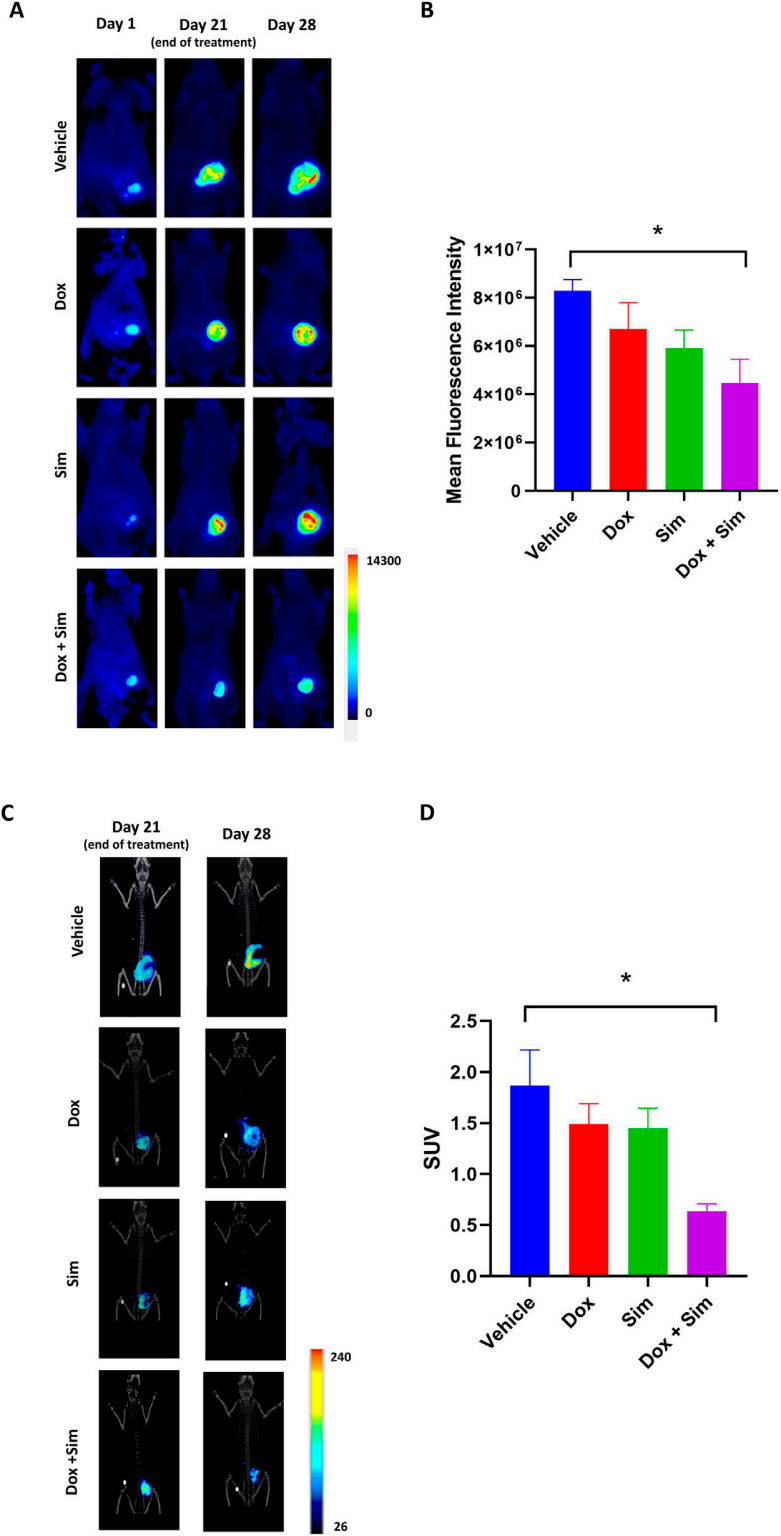
*In vivo* molecular imaging of tumor response to treatments. **(A)** Representative Fluorescence images of MDA-MB-231 cells transfected with piRFP after different treatments (n = 5). **(B)** Fluorescence intensity of each group (Vehicle, doxorubicin, simvastatin, and combination group doxorubicin with simvastatin) at day 28. Data are presented as mean ± SEM of four to five animals (*) indicates a significant difference (p < 0.05) between the vehicle vs. the combination group (Dox + Sim). **(C)** Representative PET/CT images showing 18F-FDG primary tumor uptake of different treatments. **(D)** Metabolic activity of tumors, for the different experimental groups at day 28. Data are presented as mean ± SEM of three animals (*) indicates a significant difference between the vehicle vs. the combination group (Dox + Sim).

The effects of doxorubicin and simvastatin on tumor growth correlate with NIRF images, with lower fluorescence intensity in tumors from the drug combination group on days 21 and 28, indicating less tumor growth than with individual treatments (Figure 5A). Quantification of tumor fluorescence intensity (Figure 5B) shows a significant reduction in signal in the combination group compared with the vehicle group. To further assess metabolic activity and viability within the tumors [^18^F]FDG microPET/CT imaging was performed at later stages of tumor development (Figure 5C). In the vehicle group, microPET images showed strong peripheral FDG uptake and a central hypometabolic area, suggestive of necrosis within the tumor core. In contrast, the Dox + Sim group showed a lower tumor burden and decreased metabolic activity. These findings confirm the enhanced antitumor efficacy of the combination treatment. Quantitative analysis of the standardized uptake value (SUV) (Figure 5D) confirmed decreased glucose metabolism in the combination group, supporting the enhanced therapeutic efficacy of dox + sim treatment.

### 3.6 Effect of doxorubicin and simvastatin on lung metastasis in an orthotopic TNBC model

**FIGURE 6:**
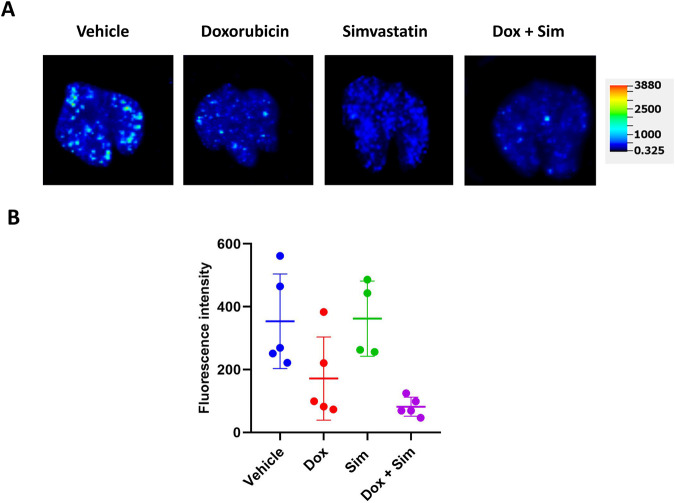
Effect of doxorubicin and simvastatin on lung metastasis. **(A)** Representative near-infrared fluorescence (NIRF) images of *ex vivo* studies of lung tissues from different treatments: vehicle, doxorubicin (1 mg/kg), simvastatin (20 mg/kg), and the combination of doxorubicin and simvastatin. **(B)** Quantification of fluorescence intensity in lung metastases from different groups. Data are presented as mean ± SD of four to five animals.

The *ex vivo* fluorescence imaging of the lungs was performed 2 weeks after the treatments were finished to study pulmonary metastasis. As shown in Figure 6, a fluorescence signal was observed in the vehicle group. This signal was reduced in the doxorubicin-treated group. In contrast, no significant change in fluorescence intensity was observed in the simvastatin-treated group compared to the control. However, a marked reduction in fluorescence signal was observed in the combination treatment group, indicating a significant decrease in pulmonary metastasis. Quantitative fluorescence analysis further confirmed a reduction of pulmonary metastasis in the combination treatment group compared to the control and individual treatment groups (Figure 6B).

**FIGURE 7:**
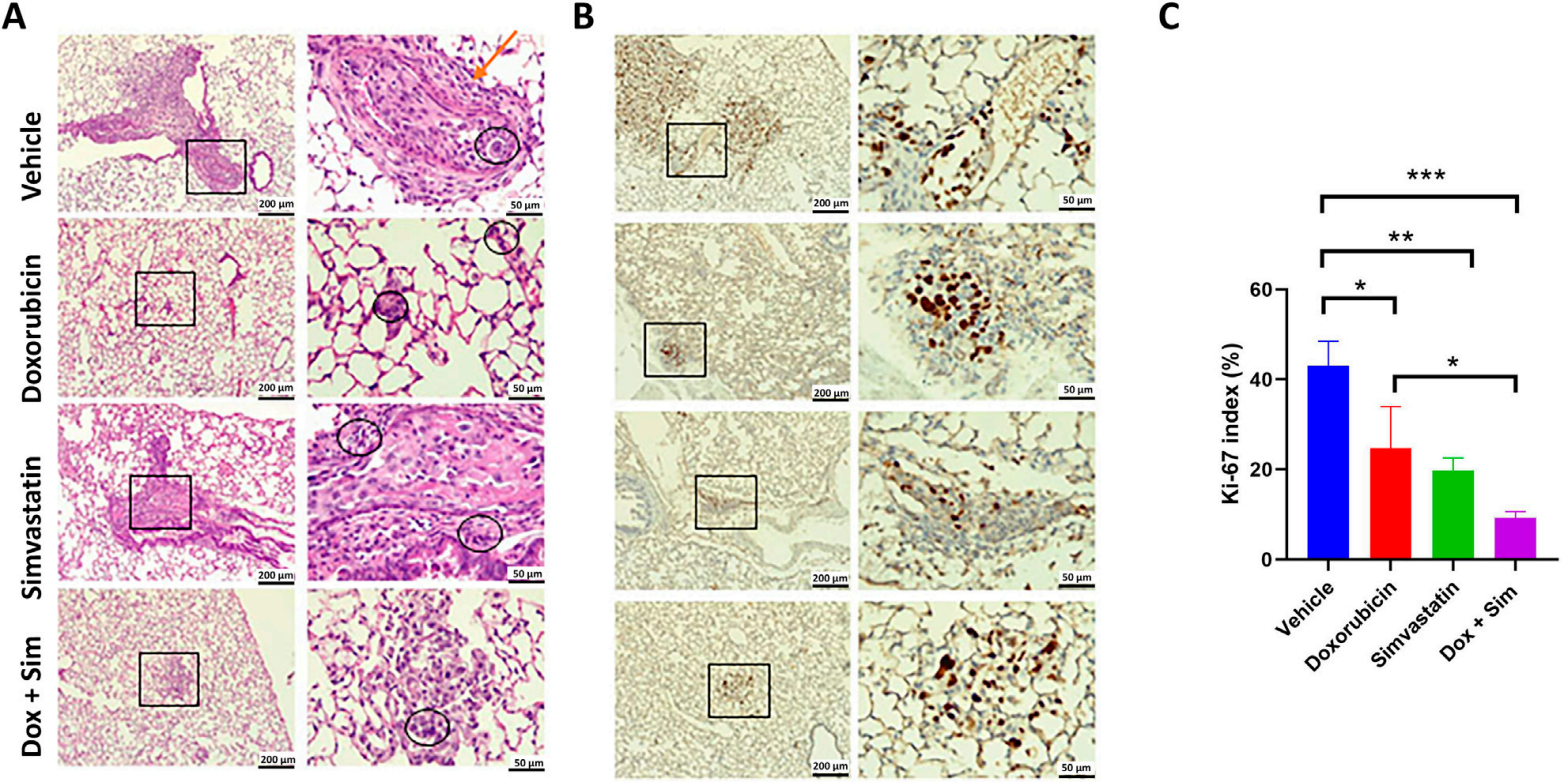
Histopathological examination of lung tissue. **(A)** Representative histopathological examination of the lungs by H&E staining analysis (n = 3). The boxed areas are shown at higher magnification (×10 to 40x) in the right H&E image. Neoplastic cells (black circled), vascular permeation (orange arrows). **(B)** Immunohistochemistry analysis. Tumor slides were immunostained with anti-Ki-67. The brown dye-stained cells represent positive cells. The boxed areas are shown at higher magnification (×10 to 40x) in the right Ki67 image (n = 3). **(C)** Quantification of Ki-67 proliferation index expressed as the percentage of Ki-67 positive nuclei relative to total nuclei. Data are presented as mean ± SD (n = 3). Statistical significance was determined using one-way ANOVA followed by Tukey’s *post hoc* test (*p < 0.05, **p < 0.01, ***p < 0.001).

The metastasis was additionally confirmed by H&E staining analysis. The H&E analysis of lung histological sections revealed significant differences among the treatments (Figure 7A). In the vehicle group, neoplastic cells were observed inside blood vessels, extending into the adjacent lung tissue, with lymphocytes interspersed among them. On the other hand, animals treated with the individual drugs showed a partial reduction in neoplastic cells in the lung parenchyma and blood vessels. However, the group treated with the combination of doxorubicin and simvastatin exhibited a significant decrease in the density of neoplastic cells and vascular and lymphatic infiltration. Immunohistochemical staining was employed to detect the presence of Ki-67. Figure 7B shows representative images of the different treatments. The vehicle group displayed a high density of Ki-67-positive cells, indicating high proliferative activity. In contrast, the groups treated with doxorubicin or simvastatin individually showed a reduction in the number of Ki-67-positive cells. However, this decrease in proliferative activity was more pronounced in the combination treatment of doxorubicin and simvastatin. Quantitative analysis confirmed a significant reduction in the Ki-67 proliferation index in the combination group compared to control and single-treatment groups (Figure 7C).

## 4 Discussion

TNBC is the most aggressive subtype of breast cancer due to the low expression of estrogen receptors (ER), progesterone receptors (PR), and human epidermal growth factor receptor 2 (HER2), which limits the effectiveness of hormone and targeted therapies. Therefore, no standard treatment protocol has been established, and therapeutic strategies are based on adjuvant and neoadjuvant chemotherapy. However, it has been reported that TNBC patients develop drug resistance and distant metastases, significantly reducing overall survival (Dent et al., 2007; Denkert et al., 2017; Nedeljkovic and Damjanovic, 2019). Therefore, it is crucial to develop new therapeutic strategies that are more effective and less toxic.

The development of new drugs has multiple challenges, such as high economic investment and long development times. In this context, drug repurposing has emerged as a promising strategy to reutilize previously approved drugs for new therapeutic indications, reducing costs and accelerating clinical application. In this study, we evaluated the potential of simvastatin, an inhibitor of 3-hydroxy-3-methylglutaryl-coenzyme A (HMG-CoA) reductase, in combination with doxorubicin, analyzing their individual and combined effects on cell viability and migration in the MDA-MB-231 cell line, as well as their impact on tumor progression and lung metastasis in an orthotopic TNBC model.

Although simvastatin is a widely used drug to lower cholesterol levels, various studies have demonstrated its potential as an antitumor agent. Its cytotoxic activity has been reported in several types of cancer, including breast, colon, pancreas, prostate, and leukemia (Cardwell et al., 2015; Li et al., 2016; Chen et al., 2018; Tian et al., 2021; Chen et al., 2022). Also, simvastatin has been reported to promote apoptosis by reducing the expression of anti-apoptotic proteins Bcl-2 and Bcl-xL and increasing the expression of pro-apoptotic proteins such as Bax, Bad, caspase-3, -8, and -9, as well as inducing the production of reactive oxygen species (ROS) (Goc et al., 2012; Spampanato et al., 2012; Xie et al., 2023). In this context, the combination of simvastatin with doxorubicin could represent a promising therapeutic strategy to enhance treatment response in TNBC.

### 4.1 *In vitro* effects of simvastatin and doxorubicin combination

Cytotoxicity assays in two-dimensional (2D) culture demonstrated that simvastatin reduces cell viability dose-dependently. Moreover, a significant decrease in the IC50 was observed, indicating that the potency of doxorubicin is greater when combined with simvastatin. Additionally, in 2D and three-dimensional (3D) cultures, we observed that simvastatin in combination with doxorubicin resulted in a significantly greater decrease in cell viability compared to individual treatments. These findings are consistent with previous studies reporting that simvastatin enhances doxorubicin cytotoxicity in MCF-7 breast cancer cells by inhibiting the RAC1 pathway and activating caspase-3 and cytochrome C (Buranrat et al., 2017).

On the other hand, O’Grady et al. demonstrated that statins can modulate the response of tumor cells to chemotherapy by inhibiting the mevalonate pathway, reducing cell proliferation, and inducing apoptosis in breast cancer cells. These mechanisms could contribute to the increased toxicity observed with the combination of simvastatin and doxorubicin (O'Grady et al., 2022). Additionally, simvastatin has been reported to have a chemosensitizing effect by inhibiting the synthesis of P-gp, an efflux transporter that reduces the intracellular accumulation of cytotoxic drugs (Zhang et al., 2016). The inhibition of P-gp increases the intracellular concentration of doxorubicin, thereby improving its therapeutic efficacy.

### 4.2 Effect on cell migration and invasion

Migration and invasion assays demonstrated that the combination of simvastatin and doxorubicin was more effective in inhibiting MDA-MB-231 cell migration compared to individual treatments. In recent years, several mechanisms have been reported that could contribute to inhibiting cell migration and invasion mediated by statins and doxorubicin. Statins have been reported to alter cytoskeletal reorganization and focal adhesion, both essential processes in cell migration (Denoyelle et al., 2001). Another mechanism reported in cell migration inhibition is the deregulation of small GTPases such as Ras and RhoA, key proteins in cytoskeletal dynamics and cell motility (Denoyelle et al., 2001; Lee et al., 2023).

Furthermore, inhibition of the mevalonate pathway affects the stability of membrane-anchored proteins, interfering with integrin signaling and cell adhesion; thereby, contributing to reduced migration and invasion (Centonze et al., 2023; Wang et al., 2024). Additionally, it has been reported that simvastatin inhibits the migration of TNBC cells by reducing the expression of extracellular matrix metalloproteinases (MMPs), particularly MMP-2 and MMP-9 (Yin et al., 2020). Together, all these mechanisms could contribute to the decrease in cell migration and invasion with the combination of doxorubicin and simvastatin.

### 4.3 *In vivo* effects on tumor growth and lung metastasis

In the orthotopic TNBC model, the combination of simvastatin and doxorubicin significantly reduced tumor growth rate compared to individual treatments. Doxorubicin has been reported to exert its antitumor effect through DNA intercalation, ROS generation, and topoisomerase II inhibition (Thorn et al., 2011; Kciuk et al., 2023). On the other hand, Simvastatin has demonstrated the ability to inhibit tumor growth in several types of cancer. Sandoval-Usme et al. reported that simvastatin inhibits proliferation, migration, and invasion in UMR-106 cells (osteosarcoma cells) by modulating JAK2 and STAT5 phosphorylation (Sandoval-Usme et al., 2014). Wang, S.T. et al. Described that simvastatin decreased tumor growth in a hepatocellular carcinoma model, and this effect was associated with increased expression of p21 and p27, AMPK activation, and inhibition of the STAT3/Skp2 pathway (Wang et al., 2017). Therefore, Simvastatin could be a promising candidate for combination therapies by enhancing tumor growth inhibition in TNBC.

The decrease in the growth rate of the primary tumor was confirmed through measurements of tumor volume, NIRF images, and multimodal imaging (microPET/CT). The combination treatment showed less tumor growth compared to individual treatments. In the vehicle group, the fluorescence signal remained homogeneous and persistent throughout the study. However, PET/CT imaging revealed a pattern of intense FDG uptake at the tumor periphery, with a central hypometabolic region on days 21 and 28, suggestive of intratumoral necrosis. This discrepancy may be attributed to the planar nature of fluorescence imaging, which integrates all emitted signals into a single 2D projection. In contrast, microPET/CT provides a three-dimensional assessment. These observations underscore the importance of complementary imaging techniques in accurately assessing tumor physiology and treatment response.

Although no evident clinical signs of systemic toxicity were observed during the study, and body weight had no under 20% in any group, an evaluation of cardiotoxicity was not performed. Future studies should incorporate detailed cardiac assessments, including histopathology and biochemical markers. However, cardiotoxicity is a well-recognized side effect of a wide range of anticancer agents, including anthracyclines such as doxorubicin. It may necessitate dose reduction, treatment interruption, or discontinuation depending on the severity of cardiac damage (Tana et al., 2026). However, simvastatin, along with other statins, has been proposed as a potential strategy to mitigate anthracycline-induced cardiotoxicity by significantly reducing the decline in left ventricular ejection fraction (LVEF) (Obasi et al., 2021). The mechanisms underlying the cardioprotective effects of simvastatin are not yet fully understood; however, accumulating evidence suggests that these effects of simvastatin may be related to its ability to reduce endothelial damage, as well as its anti-inflammatory, antioxidant, antifibrotic, and immunomodulatory properties (Stein et al., 2015; German and Liao, 2023; Jiang et al., 2021). Nevertheless, further studies are required to elucidate the cellular and molecular mechanisms involved in the cardioprotective role of statins.

The impact of the combination on lung metastasis was evaluated using NIRF imaging, which revealed a significant reduction in fluorescence intensity in animals treated with Simvastatin and doxorubicin. Histopathological analysis confirmed these results, which showed fewer tumor cells in the lung tissue of animals that received the combined treatment than those with the individual treatments.

The inhibition of lung metastasis by simvastatin may be associated with interference with cell adhesion and tumor invasion by modulating the AKT/mTOR pathway. Fang et al. reported that Simvastatin inhibits renal cancer cell growth and metastasis via the AKT/mTOR, ERK, and JAK2/STAT3 pathways (Fang et al., 2013). Stat proteins are a family of cytoplasmic transcription factors that are induced in response to stimulation by cytokines and growth factors. Many human malignancies maintain constitutively active Stat3, a member of the Stat protein family. Constitutively active Stat3 serves as an important mechanism to support the progression of cancer (Chen et al., 2024). Consequently, Stat3 pathway inhibition has been shown as an important target for the development of new anticancer therapeutic drugs, since improved efficacy against human TNBC xenografts in mice. That´s the case of simvastatin, or even other STAT3 inhibitors, such as azetidine-based small molecules, which directly inhibit Stat3 activity (Yue et al., 2022), and could explain the effect between simvastatin and doxorubicin we showed.

It was also described that simvastatin inhibited BMP-2 (Bone Morphogenetic Protein 2) activity in breast cancer cell lines. It has been reported that BMP-2, a member of the transforming growth factor-β (TGF-β) superfamily, enhances the migratory and invasive potential of MDA-MB-231 and MCF-1 breast cancer cells, likely through the upregulation of DNMT1 (DNA (cytosine-5-)-methyltransferase 1) and cholesterol regulatory genes (Yadav et al., 2023).

Additionally, Tate et al. reported that simvastatin induces the loss of actin cytoskeleton integrity and decreases cell motility. Through these mechanisms, simvastatin may contribute to reduced metastasis (Tate et al., 2017). Although the combination of simvastatin and doxorubicin showed promising results in reducing tumor progression and metastasis, the underlying molecular mechanisms were not directly investigated in this study. Further studies are required to elucidate the molecular pathways involved, including apoptosis, cell cycle regulation, and DNA damage response.

The enhanced antitumor activity observed with the combination of simvastatin and doxorubicin *in vitro* was further supported by the *in vivo* findings, where the combined treatment significantly reduced tumor growth and lung metastasis compared with monotherapy. Importantly, in this study, the doses used of both drugs in the murine orthotopic model were selected based on previous reports. The simvastatin dose of 20 mg/kg was in accordance with previous preclinical studies reported in the literature, which report efficacy and acceptable tolerability in murine breast cancer models (Watson et al., 2022). Importantly, doses within the range of 10–60 mg/kg have been commonly employed in breast cancer mouse models, including orthotopic and xenograft TNBC, with effects on tumor growth, without evidence of severe systemic toxicity (Karimi et al., 2019). Therefore, we selected a 20 mg/kg dose as an intermediate pharmacologically active dose within the effective preclinical range reported in the literature. Furthermore, the clinically used dose of simvastatin in humans is 40–80 mg/day (0.67–1.4 mg/kg) for a 60-kg adult, which corresponds to approximately 8–16 mg/kg in mice based on the Km factor method (Reagan-Shaw et al., 2008). This exposure (20 mg/kg used in the study) is close to the highest clinically used doses of simvastatin in humans (80 mg/day). Thus, the selected murine doses of simvastatin remain within a clinically relevant translational window.

For doxorubicin in mice, doses ranging from 1 to 10 mg/kg have been reported, depending on the treatment schedule and route of administration (Mabrouk et al., 2023). The dose of 1 mg/kg of doxorubicin, twice weekly for 3 weeks, was used in this study. The use of 1 mg/kg in the orthotopic TNBC model was considered appropriate to minimize severe toxicity; we did not observe body weight loss and treatment-related mortality. From a translational perspective, based on the Km factor method, a murine dose of 1 mg/kg corresponds to an approximate human equivalent dose of 0.081 mg/kg (5 mg for a 60 kg adult). Although this dose appears lower than conventional clinical doses of doxorubicin (60–75 mg/m^2^ = 97–121 mg for a 60 kg adult), it is important to consider that murine dosing schedules are repeated over shorter experimental periods, and high doses can lead to cardiotoxicity and mortality. We used the low dose of doxorubicin to evaluate the combination therapy with simvastatin without inducing toxicity that could confound the interpretation of therapeutic outcomes. However, several translational limitations should be recognized. Murine models do not recapitulate human pharmacokinetics, tumor heterogeneity, immune microenvironment complexity, differences in metabolism, and drug clearance between mice and humans, complicating direct extrapolation. Therefore, although the selected dose of both drugs is supported by preclinical literature and translational dose conversion methodologies, further pharmacokinetic and clinical studies are required to define the optimal therapeutic regimen for simvastatin as an adjuvant strategy with doxorubicin in TNBC.

In the context of current clinical practice, the advent of biological agents, particularly PD-1 inhibitors, has become part of the standard first-line treatment for TNBC patients with PD–L1–positive early-stage (Schmid et al., 2020; Schmid et al., 2024), advanced and metastatic (Im et al., 2024). Pembrolizumab, an anti-PD-1, has become an important component of standard treatment for patients with high-risk early-stage TNBC, particularly based on the results of the KEYNOTE-522 and KEYNOTE-355 trials, which demonstrated clinical benefit in progression-free and overall survival when pembrolizumab was combined with chemotherapy neoadjuvant. Consequently, pembrolizumab plus chemotherapy is currently recommended as a standard regimen for previously untreated PD-L1–positive TNBC patients. However, this therapeutic strategy does not benefit all patients. Based on the published results of KEYNOTE-522, the pathological complete response (pCR) was approximately 64.8% with pembrolizumab plus chemotherapy, indicating that around 35% of patients treated with pembrolizumab will not achieve a pCR, and 23% must discontinue treatment due to toxicity (Schmid et al., 2020; Schmid et al., 2024). For these patients, optimizing cytotoxic regimens with doxorubicin and incorporating agents such as simvastatin represent promising approaches to improving therapeutic efficacy and overcoming biological resistance. In these clinical scenarios, conventional cytotoxic drugs remain important therapeutic alternatives because of their well-established antitumor activity in TNBC and their continued use in patients with immunotherapy-resistant disease or contraindications to pembrolizumab-based regimens. Therefore, simvastatin could be a promising drug candidate that warrants further investigation, particularly for patients with immunotherapy-resistant TNBC. Furthermore, the favorable safety profile, well-known clinical use, and low cost of simvastatin support its potential translational applications as an adjuvant agent in resistant TNBC.

## 5 Conclusion

Our results demonstrated that the addition of simvastatin increased the potency of doxorubicin *in vitro* (2D cell culture and 3D spheroids). Furthermore, the combination of drugs decreased cell migration and invasion, and significantly inhibited tumor growth and lung metastatic progression in the orthotopic TNBC model. These findings suggest that simvastatin may potentiate the effect of doxorubicin and may represent a valuable adjuvant strategy for controlling metastatic progression in TNBC. However, further studies are needed to identify the precise molecular pathways involved and to assess their potential in clinical trials.

## Data Availability

The raw data supporting the conclusions of this article will be made available by the authors, without undue reservation.
